# Using Zinc Finger Nuclease Technology to Generate CRX‐Reporter Human Embryonic Stem Cells as a Tool to Identify and Study the Emergence of Photoreceptors Precursors During Pluripotent Stem Cell Differentiation

**DOI:** 10.1002/stem.2240

**Published:** 2015-11-26

**Authors:** Joseph Collin, Carla B Mellough, Birthe Dorgau, Stefan Przyborski, Inmaculada Moreno‐Gimeno, Majlinda Lako

**Affiliations:** ^1^ Institute of Genetic Medicine, Newcastle UniversityNewcastleUnited Kingdom; ^2^ School of Biological Sciences, Durham UniversityDurhamUnited Kingdom; ^3^ Centro de Investigacion Principe FelipeValenciaSpain

**Keywords:** Zinc finger nucleases, Cone‐rod homeobox, Human embryonic stem cells, Photoreceptor precursors

## Abstract

The purpose of this study was to generate human embryonic stem cell (hESC) lines harboring the green fluorescent protein (GFP) reporter at the endogenous loci of the Cone‐Rod Homeobox (*CRX*) gene, a key transcription factor in retinal development. Zinc finger nucleases (ZFNs) designed to cleave in the 3′ UTR of *CRX* were transfected into hESCs along with a donor construct containing homology to the target region, eGFP reporter, and a puromycin selection cassette. Following selection, polymerase chain reaction (PCR) and sequencing analysis of antibiotic resistant clones indicated targeted integration of the reporter cassette at the 3′ of the *CRX* gene, generating a CRX‐GFP fusion. Further analysis of a clone exhibiting homozygote integration of the GFP reporter was conducted suggesting genomic stability was preserved and no other copies of the targeting cassette were inserted elsewhere within the genome. This clone was selected for differentiation towards the retinal lineage. Immunocytochemistry of sections obtained from embryoid bodies and quantitative reverse transcriptase PCR of GFP positive and negative subpopulations purified by fluorescence activated cell sorting during the differentiation indicated a significant correlation between GFP and endogenous CRX expression. Furthermore, GFP expression was found in photoreceptor precursors emerging during hESC differentiation, but not in the retinal pigmented epithelium, retinal ganglion cells, or neurons of the developing inner nuclear layer. Together our data demonstrate the successful application of ZFN technology to generate CRX‐GFP labeled hESC lines, which can be used to study and isolate photoreceptor precursors during hESC differentiation. Stem Cells *2016;34:311–321*

**Significance Statement**

Degeneration of retinal pigmented epithelium (RPE) and photoreceptors are a frequent cause of vision impairment and blindness associated with many inherited retinal diseases and age related macular degeneration (AMD). Replacement of these cells with stem cell derived equivalents provides an excellent approach to preserve retinal structure, function and vision. Two clinical trials are at the moment focusing on replenishment of degenerate retina in Stargard't disease and AMD with human pluripotent stem cell derived RPE. However the same momentum has not been achieved for replacement of photoreceptor cells mainly due to our incomplete understanding of the best stage of achieving pluripotent stem cell derived photoreceptor maturation and integration and lack of cell surface markers to purify these cells at ease. For this reason, we have used zinc finger nuclease technology to introduce a reporter gene into the endogenous locus a key transcription factor, CRX, known to be expressed in postmitotic retinal photoreceptor precursors and to play a key role in photoreceptor genesis and maturation. We have generated and fully characterised this reporter labelled human pluripotent stem cell and have shown that genetic manipulation occurs only at the chosen locus and does not interfere with the maintenance of pluripotency. Furthermore, this approach has enabled us to mimic the expression of the CRX gene and to assess these cells during pluripotent stem cell differentiation. Our data strongly suggest that CRX is expressed in photoreceptor precursors during pluripotent stem cell differentiation, thus providing us with a tool to study and purify the molecular expression profile and engraftment capacity of these cells. To the best of our knowledge, this is the first fully characterised reporter stem cell line of endogenous CRX in emerging photoreceptor precursors and for this reason and others stated above should be of wide interest to the readers of Stem Cells.

## Introduction

Zinc finger nucleases (ZFNs) are designer nucleases which can be engineered to target a specific DNA sequence, offering huge potential for genetically modifying cells with complex genomes, such as mammalian cells 1. ZFNs are comprised of a DNA binding domain of zinc finger protein motifs (N terminal) fused to the *FokI* endonuclease domain (C terminal), a DNA cleaving domain which operates upon dimerization 1. ZFNs are thus designed to work as a pair; upon binding to target sites on opposing strands, they act as a heterodimer and cleave both strands of the DNA. Once the ZFN pair creates a DNA double strand break (DSB) the cell's inherent DNA repair process is stimulated. In the absence of a repair template, upto 20% of cells can be inaccurately repaired via nonhomologous end joining, resulting in the imprecise deletion or insertion of bases. In the presence of a donor template containing regions of homology to the target region, homologous recombination can occur resulting in the faithful copying of a template into the endogenous loci, enabling the incorporation of exogenous sequences inserted between two “arms” or regions of homology. While only transient expression of ZFNs is required over a brief period of in vitro culture, the resulting genetic manipulation is present for the life of the cell, avoiding the need for continued expression of a foreign transgene. The potential for gene targeting and editing in various complex genomes is therefore considerable and has been realized in multiple organisms 1.

One important application is genetic modification of human pluripotent stem cells. Gene targeting of mouse embryonic stem cells using classical homologous recombination‐based methods has been widely used, however, such conventional gene targeting approaches are not easily transferred to human embryonic stem cells (hESCs), mainly due to a substantial reduction in efficiency(approximately 10^−6^ 2). However, seminal reports have shown that ZFN technology can be used to carry out precise genome modification of hESCs and human induced pluripotent stem (hiPSC) cells with greater efficiency 3, 4. One application of ZFN technology is to aid the insertion of reporter genes into a specific locus of a genome, rather than relying on the transfection of promoter‐driven reporter genes, which often integrate at undetermined sites in the genome that are subject to silencing 5. By integrating into the endogenous loci the reporter gene is under the same transcriptional control as the gene itself and thus can give a more accurate representation of a genes activity, as well as protein localization in the case of reporter fusions. In hESCs, reporters have been used to assess the activity of pluripotency or differentiation markers, as the modification created in the hESCs will be present in the differentiating cells arising from these stem cells. Because the reporter gene should be expressed at a distinct differentiation stage, it should then be possible to use fluorescence‐activated cell sorting (FACS) to capture and purify stage‐specific cells during tissue morphogenesis. The captured cells can then be characterized to define potential stage‐specific markers, thus enabling basic biological studies of differentiation and the isolation of progenitor cells derived from human pluripotent stem cells has not previously been possible. This ability is of particular importance for the isolation of hESC‐ and hiPSC‐derived retinal progenitor cells for which unique cell surface markers are lacking, and for which there is a pressing demand for basic biological studies and cell replacement applications 6.

To date, reporter‐based studies for the selection of retinal progenitor cells have been very successful in the mouse 7, 8, and this work has shown that the ontogenetic stage of the donor cell is a critical factor for its integration into an adult retina. These studies have highlighted that cells which express *Nrl* (neural retina leucine zipper gene) during early postnatal development display a rod phenotype upon transplantation, while those isolated during the embryonic peak of cone genesis and which express *Crx* (cone‐rod homeobox gene), display a cone phenotype following transplantation with a shift towards the rod phenotype if isolated from postnatal retina 7, 8. hESC and hiPSC differentiation towards retinal lineages has undergone substantial advances with the advent of 3D strategies 9, 10, 11, 12; however, the same momentum has not been reached with regard to the successful integration of hESC‐ and hiPSC‐derived retinal progenitors transplanted into the adult retina. This could in part be due to the lack of available cell surface markers that can be used to purify desired cell types before transplantation, an incomplete understanding of the optimal ontogenetic stage at which to capture differentiating hESCs and hiPSCsto facilitate their functional integration following transplantation, or the inability of current in vitro culture conditions to generate progenitor cells that are developmentally equivalent to the *Nrl*‐ and *Crx*‐expressing cells isolated from postnatal murine retina. To begin to answer some of these questions, we set out to generate hESC lines that harbor reporter genes under the control of key transcription factors known to play an important role in retinal photoreceptor cell commitment. We chose *CRX* as a candidate for the following reasons: (a) *CRX* expression is localized to post‐mitotic photoreceptor precursors before the development of outer segments 13; (b) clear evidence from animal studies that *Crx* plays an essential role in cone and rod genesis 14; and (c) existing evidence that the gene's cis regulatory regions can direct reporter gene expression in photoreceptor cells in transgenic studies 15. Our studies reported herein show that ZFNs can accurately target the *CRX* gene 3′ UTR and enable the introduction of a GFP reporter at the 3′ terminus of this gene. Furthermore, expression of the GFP reporter mimics the expression of endogenous CRX during the differentiation of hESCs towards a retinal lineage, enabling the fluorescent labeling of CRX‐expressing cells with GFP during the differentiation process. Immunocytochemistry with various retinal markers up to day 90 of hESC differentiation indicates that *CRX* expression is observed in hESC‐derived photoreceptor precursors which have exited the cell cycle and lack the expression of well‐established markers characterizing the early eye field, retinal ganglion cells, and the inner nuclear layer. Together, these data suggest that CRX‐GFP labeled hESC lines created using ZFNs provide an excellent tool with which to study retinal development in vitro and photoreceptor precursors at various stages of differentiation, which can be utilized to help define the integration potential of hESC‐derived photoreceptor precursors in the intact and diseased retina.

## Materials and Methods

### hESC Culture and Differentiation

The H9 hESC line from WiCell Inc. was used in this study. Expansion of hESCs was performed on feeder cells as described previously 16. The differentiation of H9 CRX‐GFP reporter lines towards retinal lineages was performed at least three times under three‐dimensional (3D) culture conditions, using bacteriological Petri dishes (BD Biosciences) and IGF‐1 supplemented media as described in our recent publication 12.

### Preparation of ZFN Pair, Donor Construct, and Nucleofection

**Figure 1:**
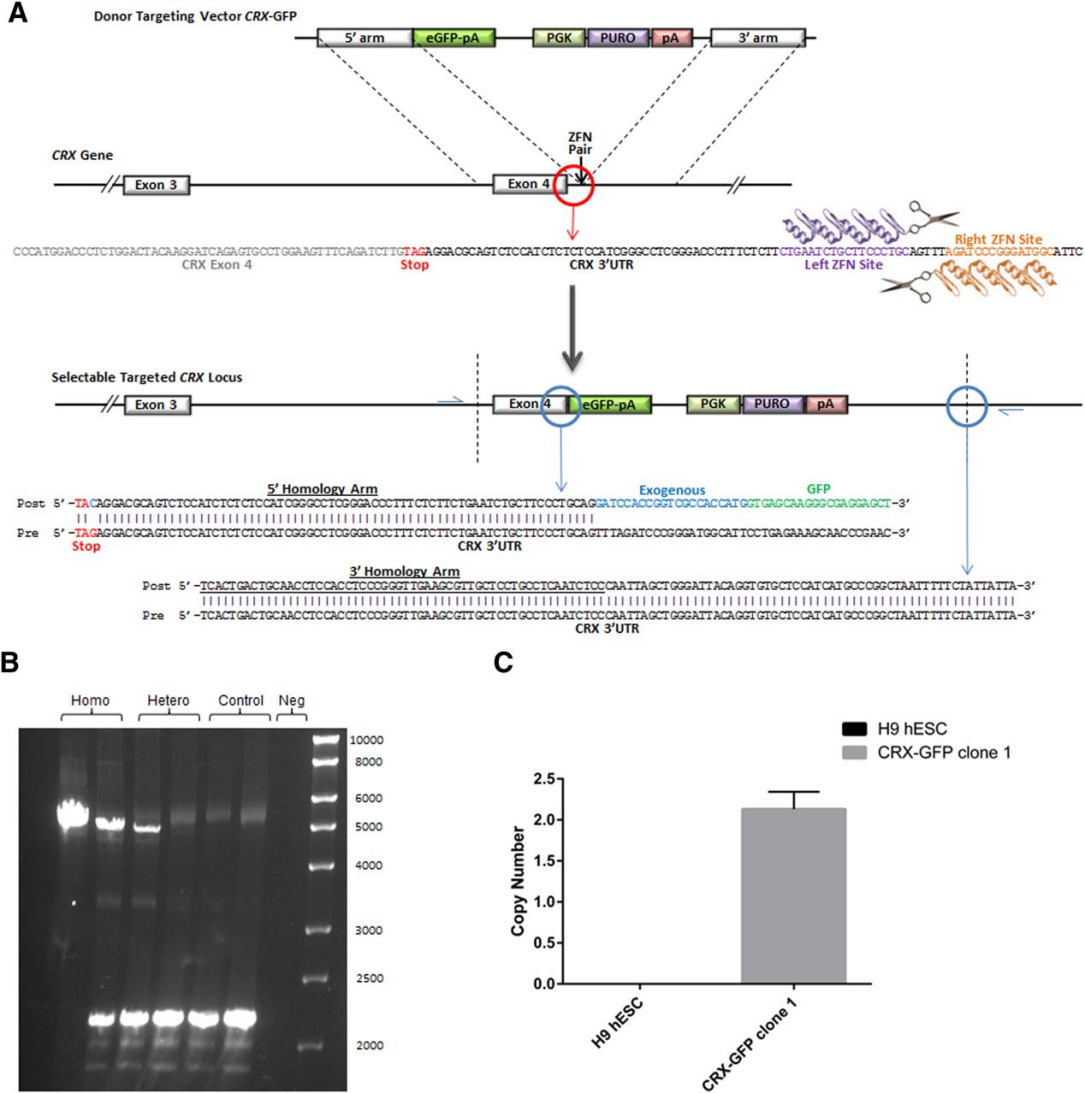
Zinc finger nuclease‐mediated targeting of green fluorescent protein (GFP) to the 3′ UTR of Cone‐rod homeobox (*CRX*). **(A)**: Schematic diagram of endogenous *CRX* gene structure, targeting cassette, targeting strategy and sequencing data from sections of the insertion site, the endogenous *CRX*‐exogenous eGFP border site and 3′ homology arm‐endogenous *CRX* border, obtained from three analyzed human embryonic stem cell (hESC) clones; **(B)**: Polymerase chain reaction (PCR) of three hESC clones with primers spanning the integration site to assess whether the cassette had integrated at the correct site. Depending on size of band produced (upper band of 5067 bp with integration, lower band of 2171 bp without) and whether the clone has a heterozygous integration (by the presence of the upper and lower bands), or a homozygote integration (a single upper band), such as clone 1 in the first lane. Lanes 2 and 3 show two clones with heterozygote integration, lanes 4–6 are genomic DNA from control untreated H9 hESCs and lane 7, a no template negative PCR control. This is a representative example of at least three repeats; **(C)**: Quantitative PCR (qPCR)‐mediated copy number analysis indicating presence of two copies of GFP (and hence targeting cassette) in CRX‐GFP hESC clone 1(c1) and its absence from wild type H9 hESC genomic DNA, data are presented as the mean ± SEM (*n* = 3). Abbreviations: CRX, cone‐rod homeobox; GFP, green fluorescent protein; hESC, human embryonic stem cell.

The ZFN pair used was designed, produced, and activity tested in K562 cells by Sigma‐Aldrich (Supporting Information Table 1; Fig. 1A). The activity in hESCs was tested following nucleofection of the ZFN pair mRNA (Supporting Information Fig. 1B). H9 cells were dissociated with Accutase (Life Technologies); 8 × 10^5^ cells then underwent nucleofection with 2 µg of each ZFN mRNA using the Human Stem Cell Nucleofector Kit 1 (Lonza) with a Nuclefector 2b device (Lonza). Following nucleofection cells were cultured as before, with the addition of Y‐27632 for 24 hours, and genomic DNA extracted (Quick‐gDNAMiniPrep, Zymo Research) 48 hours later. The frequency of cleavage products was then assessed using the SURVEYOR (CEL‐I) Mutation Detection Kit (Transgenomic), following the manufacturer's instructions) Supporting Information Fig. 1B). Briefly, polymerase chain reaction (PCR) of the region harboring the target site of the ZFN pair was performed from the genomic DNA (for primers see Supporting Information Table 1), the PCR products were denatured and then cooled to anneal oligonucleotides, followed by incubation with the CEL‐I enzyme at 42 °C for 40 minutes. The digests were then analysed by polyacrylamide gel electrophoresis and the fraction of cleaved products assessed.

The design of the donor construct was based on a published sequence by Hockemeyer et al. 3, previously used for insertion of a GFP reporter, and included 5′ and 3′ *CRX* homology arms, eGFP, and a PGK‐Puro‐pA selection cassette (Fig. 1A and full sequence in Supporting Information Fig. 2). The construct was synthesized (and cloned into pBS II SK(+)) by Eurofins Genomics. The donor DNA was prepared for transfection by using the QIAGEN Plasmid Plus Maxi kit, followed by linearization with PscI and gel purification (QIAquick Gel Extraction Kit, QIAGEN). Nucleofections were performed as before with 2.5 µg donor DNA and 2 µg of each ZFN mRNA for 8 × 10^5^ H9 cells. Forty‐eight hours post‐nucleofection, the media were supplemented with 0.5 µg/ml Puromycin for 48 hours. Emergent colonies were isolated and expanded.

### Pluripotency Assays

The expression of pluripotency markers in H9 hESCs harboring CRX‐GFP was determined using the PSC Immunocytochemistry Kit (Life Technologies), following manufacturer's guidelines. Briefly, colonies were fixed in 4% paraformaldehyde, permeabilized with 1% Saponin, blocked with 3% bovine serum albumin (BSA), incubated with antibodies against OCT4 and SSEA4 (see Supporting Information Table 3 for details) for 3 hours, washed in phosphate‐buffered saline (PBS), incubated with appropriate secondary antibodies for 1 hour, washed, incubated with 4′,6‐diamidino‐2‐phenylindole, and imaged on a Nikon A1R confocal microscope. At least 10 colonies from each clone were assessed for pluripotency.

Pluripotency of the H9 CRX‐GFP clones was then tested by spontaneous differentiation and assessment of the expression of germ layer markers identifying ectoderm (beta‐III tubulin), endoderm (alpha‐fetoprotein), and mesoderm (smooth muscle actin). Colonies were dissociated by collagenase IV (Life Technologies) and allowed to form embryoid bodies (EBs) in suspension in low‐attachment plates containing a general differentiation medium (Knockout‐Dulbecco's modified Eagle's medium, 20% fetal bovine serum, GlutaMAX, 1% non‐essential amino acids and 1% Pen‐Strep). Medium was changed daily for 7 days, EBs were then transferred onto gelatin‐coated chamber slides and cultured for a further 7 days. Presence for markers of the germ layers were then assessed using the 3‐Germ Layer Immunocytochemistry Kit (Life Technologies) following manufacturers guidelines. Briefly, colonies were fixed in 4% paraformaldehyde, permeabilized with 1% Saponin, blocked with 3% BSA, incubated with beta‐III tubulin, alpha‐fetoprotein, and smooth muscle actin antibodies (see Supporting Information Table 3 for details) for 3 hours, washed in PBS, incubated with appropriate secondary antibodies for 1 hour, washed, incubated with 4′,6‐diamidino‐2‐phenylindole, and imaged on a Nikon A1R confocal microscope. At least five EBs from each clone were assessed for markers of the three germ layers.

### PCR, Sequencing, and Copy Number Analysis

To detect the presence or absence of correctly targeted insertion of the GFP reporter cassette, PCR was performed using GoTaq Long PCR Master Mix (Promega), primers flanking the 5′ and 3′*CRX* homology arm sequences (see Supporting Information Table 1) and genomic DNA extracted (Quick‐gDNA MiniPrep, Zymo Research) from puromycin‐resistant colonies. PCR products were resolved on a 1% agarose gel. Sequencing was performed by Eurofins Genomics on genomic DNA extracted from puromycin‐resistant colonies and using primers flanking and residing across the donor construct (for primer details see Supporting Information Table 1).

Copy number analysis of GFP puromycin‐resistant clone 1 genomic DNA was carried out using a quantitative PCR (qPCR) approach with TaqMan Copy Number Assays and Genotyping Master Mix (Life Technologies) following manufacturers guidelines. Briefly, a duplex qPCR reaction was performed with an RNase P reference and GFP assay (Supporting Information Table 1) to quantify the copy number of GFP using clone 1 genomic DNA alongside untreated wild‐type H9 hESC genomic DNA. Four technical replicates were performed for each sample. Reactions were run on a 7500 Fast Real‐Time PCR System (Life Technologies) and data analyzed in Copy Caller software (Life Technologies).

### Investigation of Off‐Site ZFN Cleavage

For the prediction of potential off‐target binding sites for the 3′ CRX ZFN pair, the PROGNOS online tool 17 was used. PCR using the primers designed with this tool was then performed (for primer details see Supporting Information Table 2). PCR products were sequenced by Eurofins Genomics (for sequences see Supporting Information Table 2). Sequences were subject to BLAST analysis to confirm the presence of insertions or deletions around the predicted off‐target site.

### Immunocytochemistry

EBs resulting from the directed differentiation of hESCs towards a retinal phenotype were collected at days 30, 60, and 90 of differentiation and immunocytochemistry analysis performed on cryostat sections as described previously 16. A panel of retinal antibodies listed in Supporting Information Table 3 were used for this analysis. At least five EBs from each differentiation were sampled for immunofluorescent histochemistry. Images were obtained using a Zeiss Axio Imager.Z1 microscope with ApoTome.2 accessory equipment and AxioVision software.

### Flow Activated Cell Sorting and Quantitative RT‐PCR

EBs were disassociated into single cells using 0.05% Trypsin for 30 minutes. Trypsin was inactivated with serum‐containing media and the cells washed with PBS. Cells were sorted on a FACS Aria (BD) into GFP positive and negative fractions.RNA was extracted from each fraction (Quick‐RNA MicroPrep, Zymo Research), cDNA synthesized (High Capacity cDNA Reverse Transcription Kit, Life Technologies), preamplified (TaqMan PreAmp Master Mix, Life Technologies), and qPCR performed (TaqMan Gene Expression Master Mix, Life Technologies) using a QuantStudio7 system and software (Life Technologies). For TaqMan assays used see Supporting Information Table 1. Statistical analysis was performed using GraphPad Prism software (Version 6.05) with statistical significance tested using Student's *t*‐test.

### Karyotype Analysis

Karyotypes were determined by standard G‐banding procedure as described previously 18. At least 20 metaphases were analyzed for each sample.

### Teratoma Formation

Approximately 5–10 × 10^5^ hESC were injected subcutaneously into the right flank of in adult SCID mice and maintained for 8–12 weeks 18. All cells were co‐transplanted with 50 µl Matrigel (BD Biosciences) to enhance teratoma formation. Two to three animals were injected for each clone. After 8–12 weeks, mice were killed, tissues were dissected, fixed in Bouins overnight, processed, and sectioned according to standard procedures and counterstained with either H&E or Massons Trichrome stain. Sections (5–8 μm) were examined using bright field light microscopy and photographed as appropriate.

## Results

### Generation and Characterization of *CRX‐GFP* hESC Clones

We chose to introduce the GFP reporter at the 3′end of the *CRX* gene to avoid disruption of the coding sequence, preventing the loss of *CRX* function and maximizing the maintenance of protein localization and function. For this reason, we mutated the stop codon of *CRX* introduced in the targeting cassette, which was followed by the eGFP sequence (Fig. 1A; Supporting Information Fig. 2), thus resulting in a CRX‐GFP fusion upon ZFN pair cutting in the 3′UTR of *CRX* and insertion of the targeting cassette via homologous recombination.

Eighty two colonies arose from the initial puromycin‐treated culture following nucleofection. Of these, 24 were picked and expanded. Three of these clones were characterized for the correct integration of the targeting cassette. PCR across the integration site using primers positioned in the genomic DNA and flanking the 5′ and 3′ *CRX* homologous arms of the donor cassette was performed. This analysis indicated that one of the three selected clones, “clone 1”, harbored a homozygous integration and the other two harbored heterozygous integrations (Fig. 1B). Sequencing across the integration site was also performed, including the *CRX*‐eGFP fusion border and the homology arm‐endogenous genome border confirming correct targeted integration at the 3′ end of the *CRX* gene in each clone (Fig. 1A). All three clones were characterized for karyotypic stability (Supporting Information Fig. 3A) and maintenance of a pluripotent phenotype using in vitro assays both at the proliferative stage (Supporting Information Fig. 3B) and following differentiation towards all three germ layers (Supporting Information Fig. 3C), in addition to in vivo teratoma formation assays (Supporting Information Fig. 3D). As we set out to assess whether ZFN technology could be used to precisely tag a gene of interest with minimal disruption of the gene environment to produce an accurate reporter of gene expression, the most stringent test for this was to use a clone where homozygous tagging of the gene had occurred, and for this reason, we continued our analyses with clone 1. Further analysis to assess the genomic fidelity of clone 1 following ZFN‐mediated tagging was then conducted. Quantitative PCR based copy number analysis of GFP revealed the presence of two copies of GFP (Fig. 1C), corroborating the PCR and sequencing data and suggesting that no additional copies of GFP were present in the genome due to nontargeted insertion of the donor cassette. In addition, the 15 most likely genomic sites for targeting by the ZFN pair were predicted (Supporting Information Table 2) using the PROGNOS online tool 17. Using the primers generated from this analysis, genomic DNA from clone 1 was amplified and sequenced. This analysis indicated no insertions, deletions, or integration at predicted off‐target sites. Together our data suggest that ZFN targeting at the 3′ end of *CRX* in hESCs does not affect the maintenance of genomic stability or pluripotency.

### GFP Reporter Mimics the Expression of Endogenous CRX During Retinal Differentiation of hESC Targeted Clones

**Figure 2:**
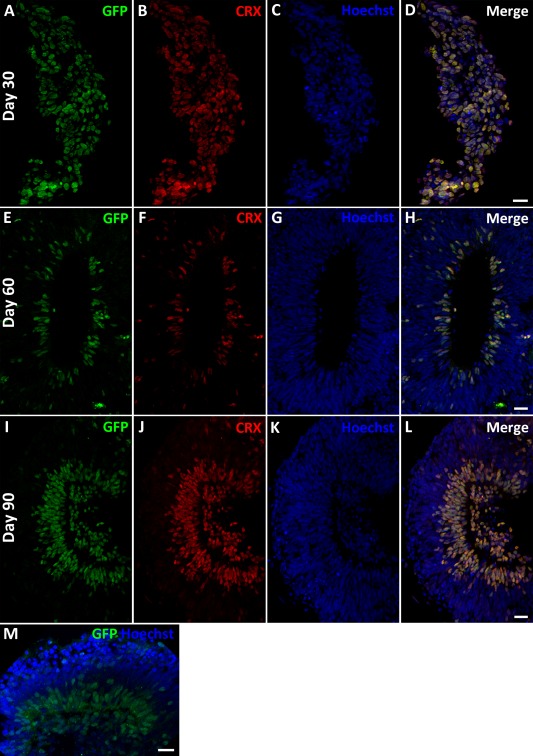
Green fluorescent protein (GFP) reporter accurately mimics the expression of endogenous cone‐rod homeobox (CRX) during human embryonic stem cell (hESC) differentiation. **(A–L)**: Immunocytochemistry with antibodies raised against CRX and GFP showing correlation at day 30 (A–D), day 60 (E–H), and day 90 (I–L) of hESC differentiation. **(M)**: Detection of GFP without antibody using confocal microscopy. Scale bars = 20 µm. Abbreviations: CRX, Cone‐rod homeobox; GFP, green fluorescent protein.

The CRX‐GFP targeted hESC clones were subjected to our 3D differentiation protocol described in Ref. 12. This protocol utilizes a single growth factor (IGF‐1) and results in formation of laminated neural retina comprising a range of well differentiated retinal phenotypes 12. This differentiation method relies on the mechanical transfer of hESC colonies to suspension conditions, forming 3D EBs which give rise to laminated neural retinal tissue displaying apically positioned photoreceptors; however, a minority of EBs shows internal cellular rosettes exhibiting a central hollow lumen with CRX positive cells found towards the internal surface. In accordance with this, we were able to detect GFP‐positive cells residing in the apical aspect of developing retinal tissue (Fig. 2A–2D) as well as the internal aspect of retinal rosettes (Fig. 2E–2L) during the 90‐day differentiation protocol.

On days 30, 60, and 90 of differentiation, floating EBs were collected and subjected to immunocytochemical analysis using antibodies directed against GFP and CRX (Fig. 2A–2L). This analysis showed an identical correlation between GFP and endogenous *CRX* expression throughout the time‐course of differentiation. Furthermore, the detection of GFP expression could be carried out without the need for an anti‐GFP antibody (Fig. 2M). Together our data suggest that a GFP reporter introduced at the 3′ terminal of *CRX* does not adversely affect the expression of *CRX* and can accurately mimic the expression of endogenous *CRX*.

### CRX/GFP Expression Is Found in Photoreceptor Precursors During hESC Differentiation

**Figure 3:**
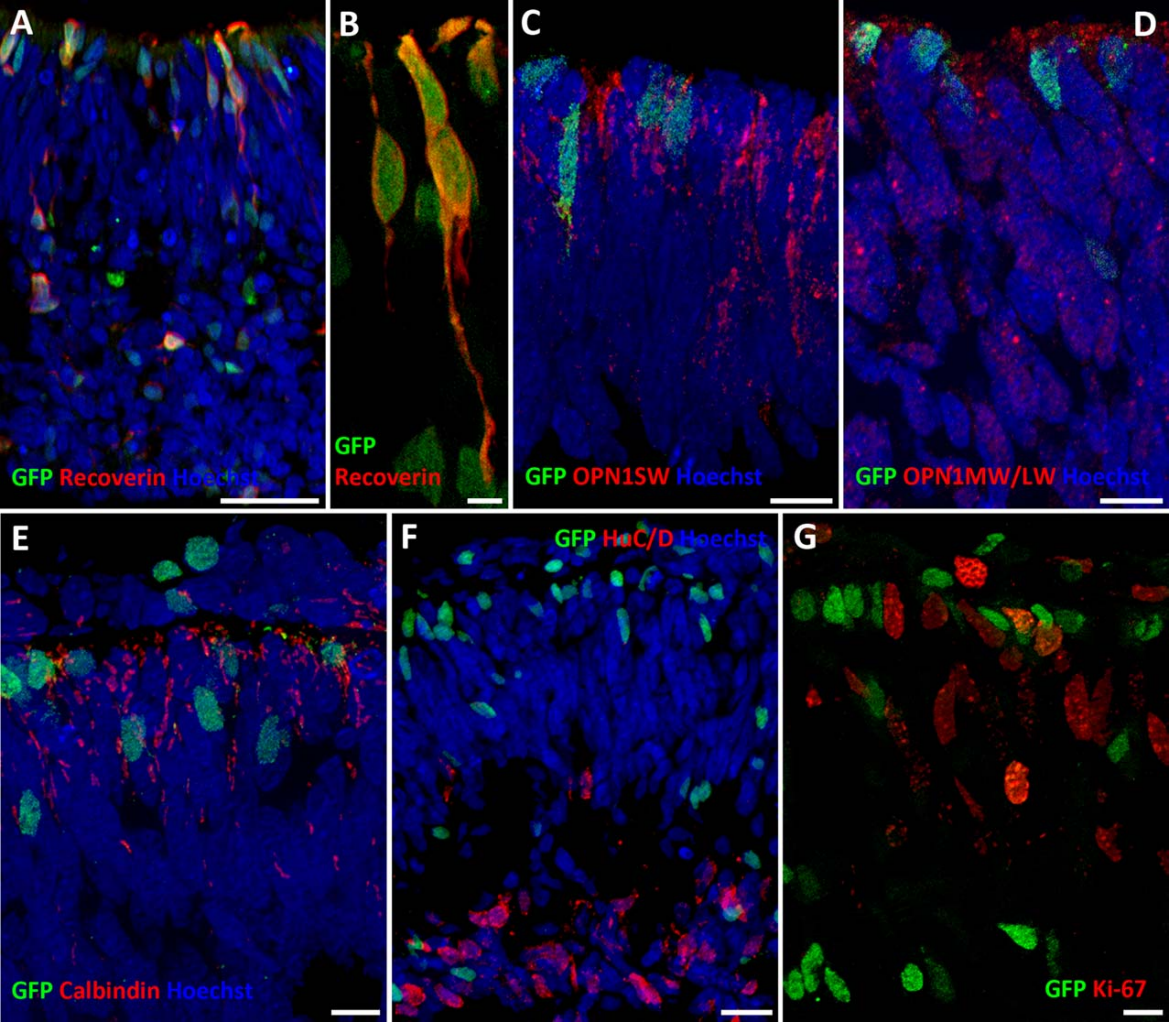
Cone‐rod homeobox (CRX)/green fluorescent protein (GFP) expression is found in photoreceptor precursors at day 60 of human embryonic stem cell (hESC) differentiation. Immunocytochemistry with antibodies raised against GFP and Recoverin **(A, B)**, OPN1SW **(C)**, OPN1MW/LW **(D)**, Calbindin **(E)**, HuC/D **(F)**, and Ki‐67 **(G)**. Scale bars = 20 µm (A, F), 5 µm (B), and 10 µm (C, D, E). Abbreviation: GFP, green fluorescent protein

**Figure 4:**
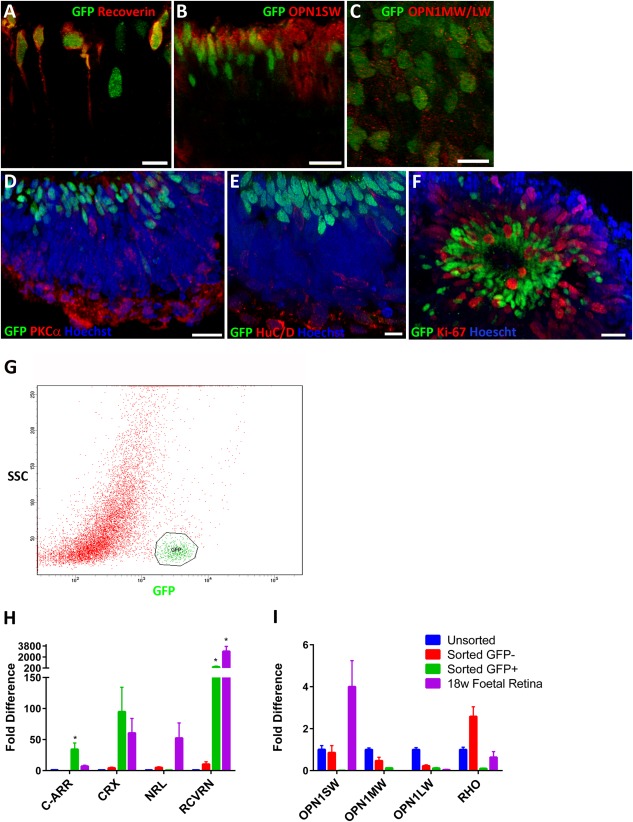
Cone‐rod homeobox (CRX) expression in photoreceptor precursors assessed by immunocytochemistry and quantitative real‐time polymerase chain reaction (qRT‐PCR) at day 90 of differentiation. Immunocytochemistry with antibodies raised against green fluorescent protein (GFP) and Recoverin **(A)**, OPN1SW **(B)**, OPN1MW/LW **(C)**, PKCα **(D)**, HuC/D **(E)**, and Ki‐67 **(F)**. Scale bars = 10 µm (E) and 20 µm (A, B, C, D, F); **(G)** Flow activated cell sorting of GFP‐positive (green) and GFP‐negative (red) populations; **(H)** and **(I)** qRT‐PCR analysis of unsorted, flow activated cell sorted GFP‐positive (Sorted GFP+) and GFP‐negative (Sorted GFP‐) populations and fetal retina at 18 weeks of gestation (18 weeks fetal retina), indicating *CRX, RECOVERIN* (*RCVRN*), and *CONE ARRESTIN* (*C‐ARR*) expression in the GFP‐positive population. Expression of short, medium, and long wave opsins (*OPN1SW, OPN1MW*, and *OPN1LW*) and rhodopsin (*RHO*) are also shown. Data are presented as the mean ± SEM (*n* = 3). Significant differences found are marked with an asterisk (*). Abbreviation: GFP, green fluorescent protein.

Throughout the differentiation period, immunocytochemical analysis was performed using markers characterizing the early stages of eyefield formation, emergence of photoreceptor precursors, mature photoreceptors, and other retinal phenotypes. Co‐immunostaining with an anti‐GFP antibody at day 60 of differentiation revealed no co‐expression with early eyefield markers (Supporting Information Fig. 4), indicating that CRX is not expressed with RAX or PAX6 during the early stages of optic cup formation. As differentiation proceeded, abundant RECOVERIN expression was observed at days 60 and 90 (Figs. 3A, 3B, 4A) in the outer nuclear layer of the developing optic cup as reported in our recent publication 12. Interestingly, all RECOVERIN‐positive cells co‐expressed CRX, while only 70% of CRX‐expressing cells were double‐labeled with RECOVERIN (data not shown). A similar expression pattern was observed in the developing human retina at 16 and 18 weeks of gestation (Supporting Information Fig. 5A–5D). Furthermore, there was no overlap in the expression of GFP with Ki67, suggesting that the CRX‐expressing cells were nonproliferative (Figs. 3G, 4F). In addition, no CRX expression was observed in the emerging retinal pigmented epithelium layer (data not shown) over the differentiation period.

Expression of cone photoreceptor markers (OPN1SW and OPN1MW+OPN1LW) was tested at days 60 and 90 of differentiation and a punctate cytoplasmic expression pattern was observed (Figs. 3C, 3D, 4B, 4C), perhaps indicative of very early stages of cone photoreceptor genesis which lacks the typical plasma membrane localization of Opsins reported at later stages of differentiation 11. Similar findings were reported by Kaewkhaw et al. 19 who noted minimal short wave opsin and no rhodopsin or medium and long wave opsin expression at day 90 of hESC differentiation. Double‐labeling of tissue with GFP and cone photoreceptor markers (Opsin Blue/OPN1SW and Opsin Red+Green/OPN1MW+OPN1LW, Figs. 3C, 3D, 4B, 4C) or Calbindin (expressed in several cell types including cone photoreceptors, Fig. 3E) indicated no obvious co‐expression, suggesting a lack of CRX expression in developing cone photoreceptors emerging within hESC derived optic cups on days 60 and 90 of hESC differentiation.

No co‐expression was observed for CRX with PKCα (expressed in Rod ON Bipolar Cells, Fig. 4D) or HuC/D (expressed in amacrine and ganglion cells, Figs. 3F, 4E) or, suggesting a lack of CRX expression in retinal ganglion cells and developing inner nuclear layer retinal neurones. These results were further corroborated by FACS combined with quantitative RT‐PCR (Fig. 4G–4I) where significantly higher expression of retinal photoreceptor precursor markers *(CRX, RECOVERIN*, and *CONE ARRESTIN*) was found in the GFP positive fraction (Fig. 4H). In contrast, higher expression of markers of postmitotic rod precursors (*NRL*) (Fig. 4H), mature rods (*RHO*), and cone photoreceptors (*OPN1SW, OPN1MW, OPN1LW*) were found in the GFP negative fraction (Fig. 4I). To evaluate the developmental stage of CRX‐GFP positive cells, we performed qRT‐PCR analysis of the same markers in the developing human retina obtained at 18 weeks of gestation. *CRX* was expressed at similar levels, while *RECOVERIN* was expressed at a higher level in the developing retina when compared with the CRX‐GFP‐positive population (Fig. 4H). Conversely, *CONE ARRESTIN* was expressed at higher levels in the CRX‐GFP‐positive population (Fig. 4H). Together these data suggest that the CRX‐GFP‐positive population shows similarities to the 18 week fetal retina with high expression of photoreceptor markers but due to the greater cone expression may be at a different developmental stage or consist of an enriched precursor population, however, to fully ascertain this further investigation with expression studies at other developmental and differentiation time points would be required and are beyond the scope of this paper.

In summary, our data suggest that *CRX* expression is confined to two cell types during the first 90 days of hESC differentiation, namely *RECOVERIN*‐expressing photoreceptor precursors situated in the developing outer nuclear layer of the optic cup and in a subpopulation of retinal progenitors that appear to be nonproliferative but do not yet express any of the markers characterizing the mature retinal phenotypes that we have tested herein. Detection of *CRX* expression as well as genes activated by *CRX* (e.g., *CONE ARRESTIN*) indicates that *CRX* remains functional following the ZFN‐mediated tagging with GFP.

## Discussion

Reporter lines that facilitate the detection of emerging photoreceptors are a useful tool for monitoring and improving hESC/hiPSC differentiation protocols towards retinal lineages and also enable the selection of specific cell types, analysis of their molecular and cell surface profile and engraftment capacity. Using plasmid and lentiviral based approaches, a few reporter hESC and hiPSC lines have been generated to monitor the emergence of retinal ganglion like cells 20 and photoreceptors 21. Both of these approaches can suffer from random integration into the genome and subsequent silencing of reporter cassettes 5. Insertion of promoter‐reporter constructs into safe loci (e.g., AAVS1) has also been suggested as a potential way of circumventing the silencing of the reporter cassettes; however, this method still suffers from the limitations imposed by the selected promoter region being inserted into the cassette, which may not fully replicate the regulatory region of the endogenous gene. To bypass these issues, we have applied ZFN technology combined with the inherent repair of DNA DSBs by homologous recombination to generate a hESC line which harbors the GFP reporter as a 3′ terminal fusion of the endogenous *CRX* gene, a well described marker of postmitotic photoreceptor precursors. The reason for targeting 3′ of *CRX* was based on previous findings in the mouse system which indicated that the 5′ UTR (up to 12 kb) was not sufficient to direct high level reporter gene expression in photoreceptor cells compared with a mixture of 5′ and 3′ UTR regions in murine transgenic lines 15. Generating a 3′ reporter fusion presents several advantages, such as maintenance of the full‐length gene sequence with less likelihood of disruption of the coding sequence while maximizing the similarity of reporter expression to that of the endogenous gene, however, it also presents technical challenges related to the presence of AT‐rich sequences commonly found at the 3′ UTR 22, which are notoriously difficult to clone (and hence to construct the integrating cassette) and are also less preferable for ZFN targeting when compared with GC‐rich regions 23, 24. This potential for lower integration efficiency did not pose a problem for our study as we were able to isolate 82 clones and further characterize three CRX‐GFP targeted hESC clones (one homozygous and two heterozygous), which met the full assessment criteria for pluripotency, indicating that CRX targeting is compatible with the maintenance of pluripotency. Most importantly, no genomic abnormalities were observed after copy number analysis and sequencing of the top 15 most likely cutting sites, suggesting that the CRX‐GFP targeted clones generated in this study provide a bona fide tool for hESC retinal differentiation studies.

Since *CRX* is not expressed at the pluripotent stem cell stage, we subjected our targeted clones to our recently described 3D retinal differentiation protocol 12 and performed immunocytochemistry with antibodies against CRX and GFP during both early and late stages of differentiation. The anti‐GFP antibody was employed for co‐immunocytochemistry with antibodies against cellular markers due to the observation of reduced GFP fluorescence following processing for immunocytochemistry and to obtain comparable levels of fluorescence intensity between both antibodies allowing optimal image clarity when performing the co‐staining. With this method, we were able to show perfect correlation between the expression and localization of CRX and GFP, suggesting that the GFP reporter accurately mimics the expression of endogenous *CRX* during hESC differentiation. Importantly, we were also able to detect GFP expression without the need for immunocytochemistry indicating that GFP confers sufficient reporter activity to monitor the emergence of *CRX* expressing cells during hESC differentiation. The ability to detect CRX expression also indicated that expression of CRX had not been adversely hampered by ZFN‐mediated tagging with GFP. Furthermore, CRX has been shown to be required for the transactivation of its own gene expression and its maintenance 25; henceforth, continued detection of *CRX* mRNA and protein expression throughout our differentiation time course further reinforces the notion that ZFN‐mediated GFP tagging has not interfered with *CRX* function.

Expression studies in human fetal retina have suggested that *CRX* is expressed in photoreceptors and in the inner nuclear layer 26 and mouse transgenic studies have also shown *CRX* expression in bipolar cells in addition to photoreceptors 15, 25. To investigate the expression of *CRX* during hESC differentiation we performed immunocytochemistry with retinal precursor and retinal lineage markers. We further validated this analysis by performing quantitative RT‐PCR of GFP positive and negative populations isolated by FACS. We found that the GFP‐positive population displayed increased expression of photoreceptor markers (*CRX, RECOVERIN*, and *CONE ARRESTIN*) when compared with the GFP negative population or unsorted cells. In addition to transactivating its own expression, *CRX* has been shown to be important for activating and maintaining the expression of several key photoreceptor genes 25, 27, 28, 29, 30. For example, the *CONE ARRESTIN* gene harbors several CRX binding sites in its promoter region which are important for its activation 31, 32, 33, hence expression of *CONE ARRESTIN* in the CRX‐GFP‐positive population indicates that the CRX protein remains functional and able to activate downstream targets after ZFN‐mediated GFP tagging.

It was interesting to observe that the expression of *RHODOPSIN*, a mature rod photoreceptor marker, was greater in the GFP negative subpopulation, suggesting confined expression of the GFP reporter to photoreceptor precursors emerging during 90 days of hESC differentiation. While we could detect expression of cone opsins in a punctate pattern that may be typical of an immature phenotype, we were unable to observe mature rod markers by immunocytochemistry, which indicates either a very low frequency of rod differentiation at day 90, or that the ontogenic stage of day 90 hESC‐derived retinal tissue precedes the developmental peak of rod genesis, akin to the pattern of retinal cell emergence during human development. These data, however, should be interpreted with caution, as the expression of opsins in our study was low or of an immature nature. These findings are corroborated by a recent publication 19 and suggest that longer differentiation experiments are required to investigate CRX reporter expression in photoreceptors that display an advanced stage of morphological features and electrophysiological function. When compared with developing retina at 18 weeks of gestation, higher *CONE ARRESTIN* expression was observed in the CRX‐GFP‐positive population, perhaps indicating a more advanced stage of cone genesis in the CRX‐positive population isolated from hESC differentiation, although this needs to be further investigated using comparative analysis with retina obtained at additional developmental periods. Intriguingly NRL expression is observed in the fetal retina but is absent from the CRX‐GFP‐positive population. This together with the expression of *CONE ARRESTIN* may mark these cells as cone‐specific progenitors; however, the heterogeneous nature of the hESC differentiation could equally yield populations of varying maturity with some cells beginning to acquire a cone photoreceptor fate while other cells are at an earlier post‐mitotic photoreceptor progenitor phase and rod genesis is yet to occur.

As outlined by previous studies in the mouse model 8, we also found that CRX expression was a marker of retinal progenitor cells that had exited the cell cycle, as assessed by lack of Ki67 expression in the GFP positive subpopulation. We also found a small percentage of CRX‐positive cells that did not co‐express RECOVERIN both in hESC‐derived laminated retina and in human fetal retina. It is already known that in the developing retina progenitor cells undergo interkinetic nuclear migration, where the nucleus oscillates in an apical to basal fashion throughout the full thickness of the retinal neuroepithelium in synchrony with the cell cycle. DNA duplication occurs towards the basal surface while mitosis occurs at the apical aspect. Some of the CRX‐GFP positive cells lacking *RECOVERIN* expression were situated towards the apical surface, so it is possible that they move towards this rim to complete mitosis before acquiring RECOVERIN expression and committing to a photoreceptor fate. If this were the case, these cells should also express Ki67. However, our analysis indicated that all CRX positive cells lack Ki67 expression leaving open the question of what these cells may be. Earlier studies in mouse transgenic models have shown that CRX expression can also be detected in bipolar cells 15; however, we did not observe any CRX cells to express PKCα, ruling out the subset of bipolar cells characterized by the expression of this marker. Clearly, further studies are required to investigate this CRX population more completely; however, the GFP reporter described in this manuscript would also enable laser dissection capture of this subset of CRX positive cells and permit further analysis at the transcriptional level to help determine their identity.

## Conclusions

In conclusion we have successfully generated CRX‐GFP reporter hESC lines, which can be used to study the molecular and cell surface profile of human photoreceptor precursors and enable their isolation for transplantation studies. We hope that these hESC lines will provide a universally useful tool with which to monitor and improve differentiation protocols, discover useful cell surface markers and develop clinically applicable strategies for the purification of hESC‐derived retinal photoreceptor precursors for transplantation.

## Author Contributions

J.C.: designed and performed research, data analysis, figure preparation, manuscript writing and final approval of manuscript; C.M.: performed research, figure preparation, data analysis and final approval of manuscript; B.D.: performed data analysis, figure preparation and final approval of manuscript; S.P. and I.M.‐G.: performed data analysis and final approval of manuscript; M.L.: designed and performed research, data analysis, figure preparation, manuscript writing, fund raising and final approval of manuscript.

## Disclosure of Potential Conflicts of Interest

The authors indicate no potential conflicts of interest.

*Note added in proof*: Whilst this paper was under revision, Swaroop and colleagues 19 reported similar findings on generation of a CRX‐GFP marked hESC line, which utilized a CRX promoter based plasmid to mark the expression of emerging CRX progenitors during the retinal differentiation process.

## Supporting information

Supporting Information Figure 1

Supporting Information Figure 2

Supporting Information Figure 3

Supporting Information Figure 4

Supporting Information Table 1

Supporting Information Table 2

Supporting Information Table 3

Supporting Information

Supporting Information
