# Supplementary figures and images for: Using Zinc Finger Nuclease Technology to Generate CRX‐Reporter Human Embryonic Stem Cells as a Tool to Identify and Study the Emergence of Photoreceptors Precursors During Pluripotent Stem Cell Differentiation
Source: Stem Cells. 2015 Nov 26;34(2):311–21. doi: 10.1002/stem.2240 (PMC4832345; doi:10.1002/stem.2240)

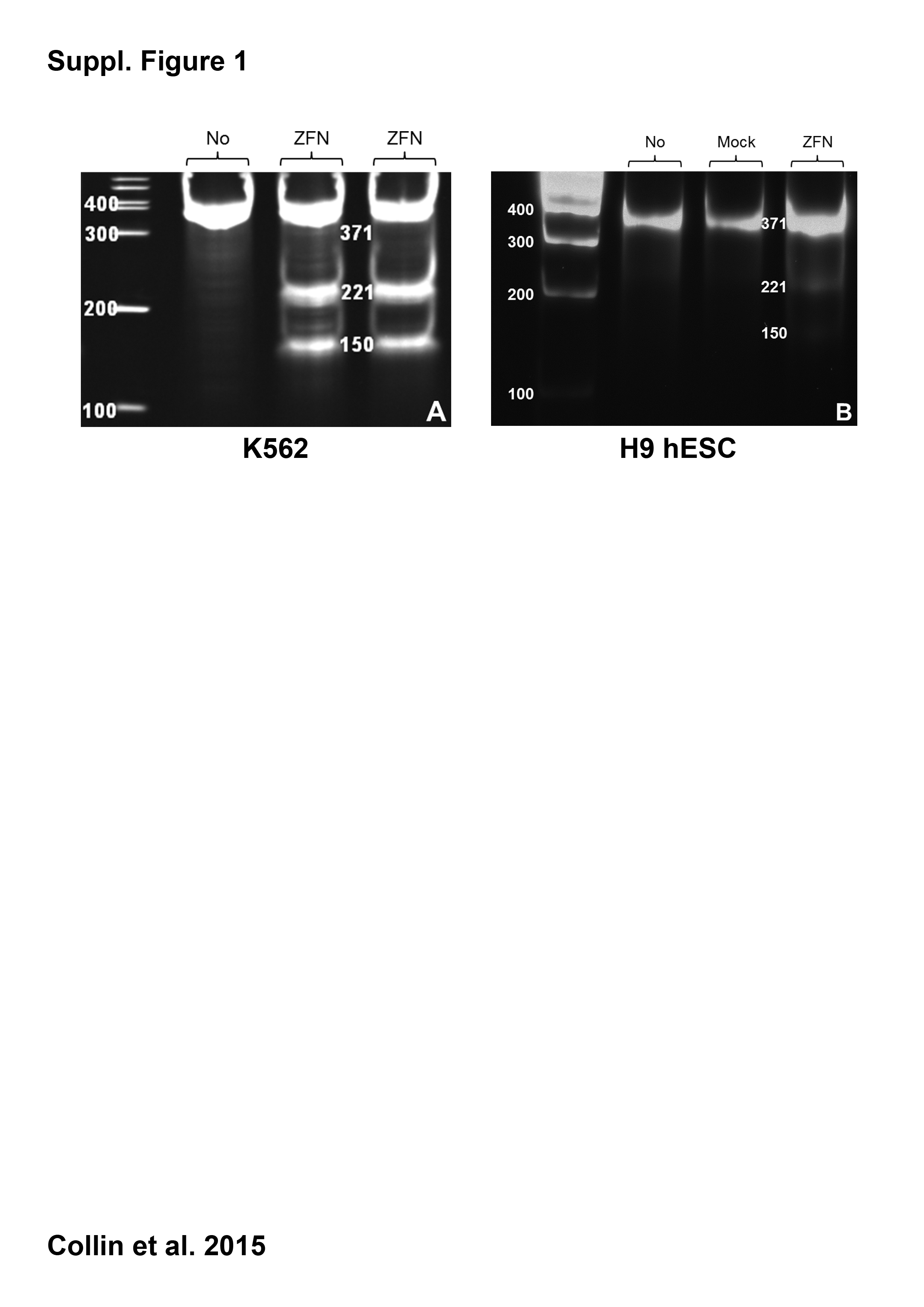

Supplement: Supplementary file 1 — Supporting Information Figure 1 [file STEM-34-311-s001.tif]

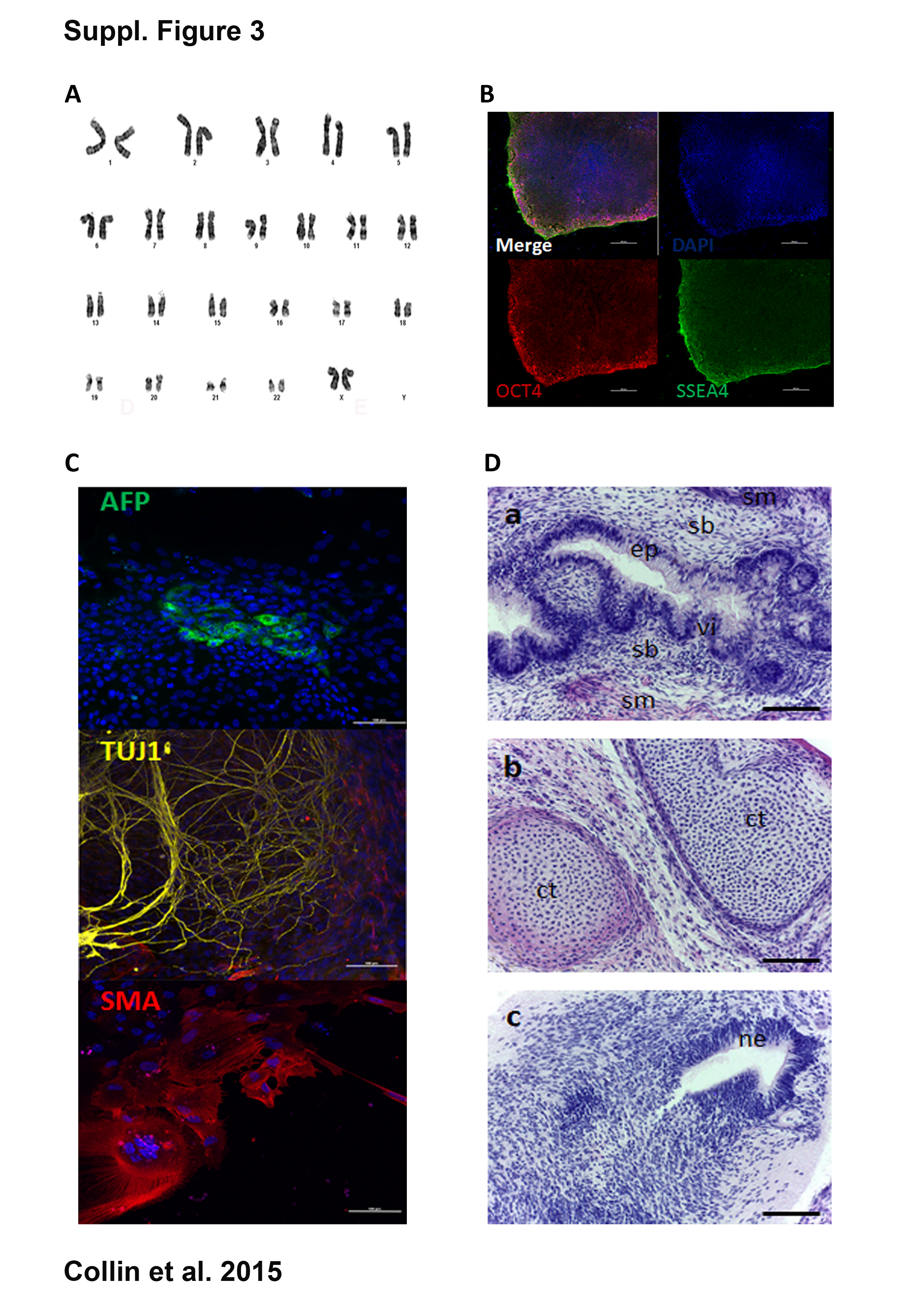

Supplement: Supplementary file 3 — Supporting Information Figure 3 [file STEM-34-311-s003.tif]

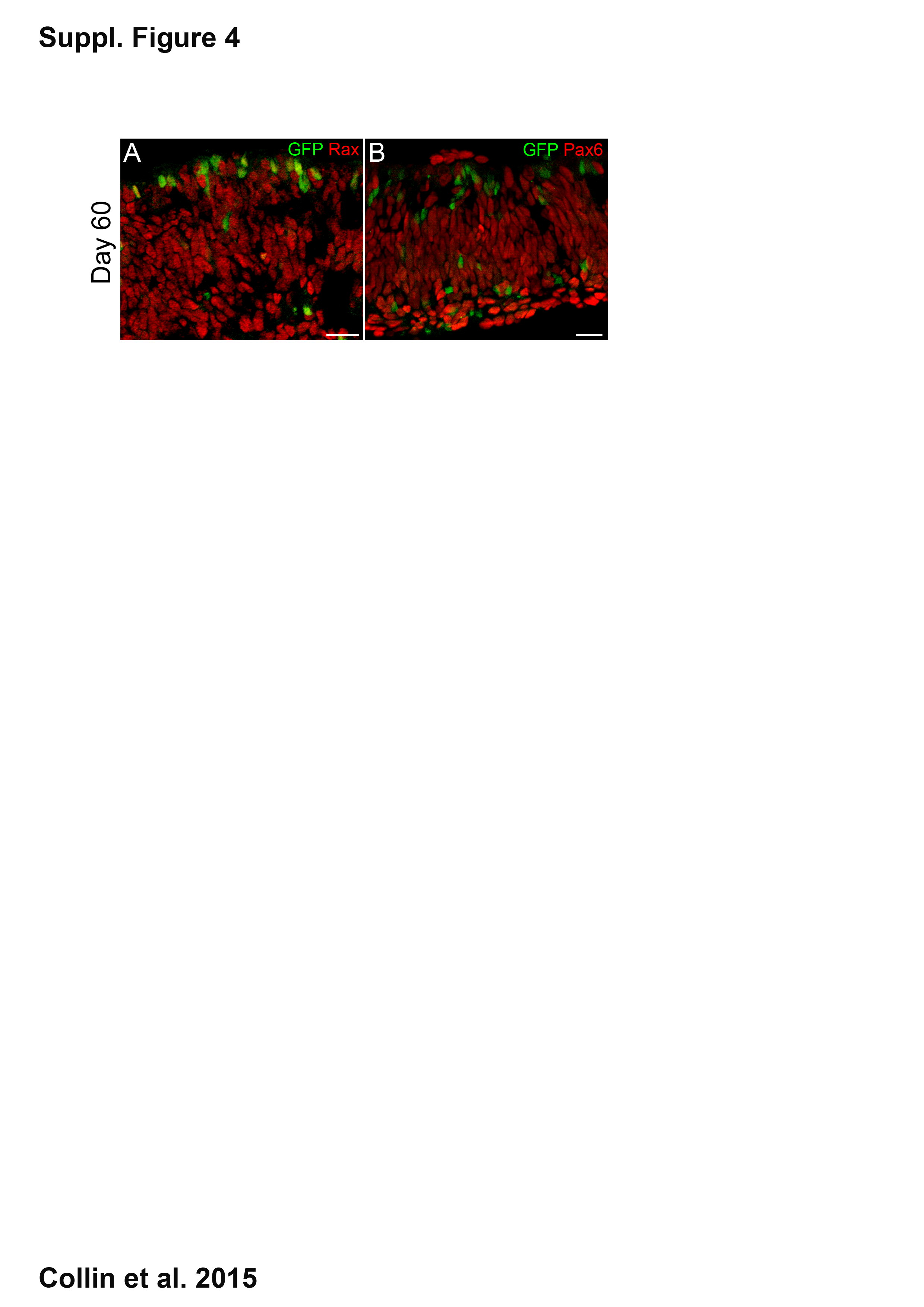

Supplement: Supplementary file 4 — Supporting Information Figure 4 [file STEM-34-311-s004.tif]
