# Supplementary material for: Using Zinc Finger Nuclease Technology to Generate CRX‐Reporter Human Embryonic Stem Cells as a Tool to Identify and Study the Emergence of Photoreceptors Precursors During Pluripotent Stem Cell Differentiation
Source: Stem Cells. 2015 Nov 26;34(2):311–21. doi: 10.1002/stem.2240 (PMC4832345; doi:10.1002/stem.2240)
Supplement: Supplementary file 2 — Supporting Information Figure 2 [file STEM-34-311-s002.docx]

**Supplementary Figure 2: CRX Donor Cassette Sequence**

GTGTCCTCATCCCCGGGCACCCTGCGGCTCTCCTGGGCCTCTTCCCCACTTACCCACCCCCATCTCCGCTCTTATCCCCCAGGTTTGGTTCAAGAACCGGAGGGCTAAATGCAGGCAGCAGCGACAGCAGCAGAAACAGCAGCAGCAGCCCCCAGGGGGCCAGGCCAAGGCCCGGCCTGCCAAGAGGAAGGCGGGCACGTCCCCAAGACCCTCCACAGATGTGTGTCCAGACCCTCTGGGCATCTCAGATTCCTACAGTCCCCCTCTGCCCGGCCCCTCAGGCTCCCCAACCACGGCAGTGGCCACTGTGTCCATCTGGAGCCCAGCCTCAGAGTCCCCTTTGCCTGAGGCGCAGCGGGCTGGGCTGGTGGCCTCAGGGCCGTCTCTGACCTCCGCCCCCTATGCCATGACCTACGCCCCGGCCTCCGCTTTCTGCTCTTCCCCCTCCGCCTATGGGTCTCCGAGCTCCTATTTCAGCGGCCTAGACCCCTACCTTTCTCCCATGGTGCCCCAGCTAGGGGGCCCGGCTCTTAGCCCCCTCTCTGGCCCCTCCGTGGGACCTTCCCTGGCCCAGTCCCCCACCTCCCTATCAGGCCAGAGCTATGGCGCCTACAGCCCCGTGGATAGCTTGGAATTCAAGGACCCCACGGGCACCTGGAAATTCACCTACAATCCCATGGACCCTCTGGACTACAAGGATCAGAGTGCCTGGAAGTTTCAGATCTTGTACAGGACGCAGTCTCCATCTCTCTCCATCGGGCCTCGGGACCCTTTCTCTTCTGAATCTGCTTCCCTGCAGgatccaccggtcgccaccatggtgagcaagggcgaggagctgttcaccggggtggtgcccatcctggtcgagctggacggcgacgtaaacggccacaagttcagcgtgtccggcgagggcgagggcgatgccacctacggcaagctgaccctgaagttcatctgcaccaccggcaagctgcccgtgccctggcccaccctcgtgaccaccctgacctacggcgtgcagtgcttcagccgctaccccgaccacatgaagcagcacgacttcttcaagtccgccatgcccgaaggctacgtccaggagcgcaccatcttcttcaaggacgacggcaactacaagacccgcgccgaggtgaagttcgagggcgacaccctggtgaaccgcatcgagctgaagggcatcgacttcaaggaggacggcaacatcctggggcacaagctggagtacaactacaacagccacaacgtctatatcatggccgacaagcagaagaacggcatcaaggtgaacttcaagatccgccacaacatcgaggacggcagcgtgcagctcgccgaccactaccagcagaacacccccatcggcgacggccccgtgctgctgcccgacaaccactacctgagcacccagtccgccctgagcaaagaccccaacgagaagcgcgatcacatggtcctgctggagttcgtgaccgccgccgggatcactctcggcatggacgagctgtacaagtaaagcggccgcgactctagatcataatcagccataccacatttgtagaggttttacttgctttaaaaaacctcccacacctccccctgaacctgaaacataaaatgaatgcaattgttgttgttaacttgtttattgcagcttataatggttacaaataaagcaatagcatcacaaatttcacaaataaagcatttttttcactgcattctagttgtggtttgtccaaactcatcaatgtatcttaagggtaccgagctcgaattccgatcatattcaataacccttaatataacttcgtataatgtatgctatacgaagttattaggtctgaagaggagtttacgtccagccaagcttaggatctcgacctcgaaattctaccgggtaggggaggcgcttttcccaaggcagtctggagcatgcgctttagcagccccgctggcacttggcgctacacaagtggcctctggcctcgcacacattccacatccaccggtagcgccaaccggctccgttctttggtggccccttcgcgccaccttctactcctcccctagtcaggaagttcccccccgccccgcagctcgcgtcgtgcaggacgtgacaaatggaagtagcacgtctcactagtctcgtgcagatggacagcaccgctgagcaatggaagcgggtaggcctttggggcagcggccaatagcagctttgctccttcgctttctgggctcagcagctgggaagggtgggtccgggggcgggctcaggggcgggctcaggggcggggcgggcgcccgaaggtcctccggaggcccggcattctgcacgcttcaaaagcgcacgtctgccgcgctgttctcctcttcctcatctccgggcctttcgacctgcatccatctagatctcgagcagctgaagcttaccatgaccgagtacaagcccacggtgcgcctcgccacccgcgacgacgtccccagggccgtacgcaccctcgccgccgcgttcgccgactaccccgccacgcgccacaccgtcgatccggaccgccacatcgagcgggtcaccgagctgcaagaactcttcctcacgcgcgtcgggctcgacatcggcaaggtgtgggtcgcggacgacggcgccgcggtggcggtctggaccacgccggagagcgtcgaagcgggggcggtgttcgccgagatcggcccgcgcatggccgagttgagcggttcccggctggccgcgcagcaacagatggaaggcctcctggcgccgcaccggcccaaggagcccgcgtggttcctggccaccgtcggcgtctcgcccgaccaccagggcaagggtctgggcagcgccgtcgtgctccccggagtggaggcggccgagcgcgccggggtgcccgccttcctggagacctccgcgccccgcaacctccccttctacgagcggctcggcttcaccgtcaccgccgacgtcgaggtgcccgaaggaccgcgcacctggtgcatgacccgcaagcccggtgcctgacgcccgccccacgacccgcagcgcccgaccgaaaggagcgcacgaccccatgcatcgatgatatcagatccccgggatgcagaaattgatgatctattaaacaataaagatgtccactaaaatggaagtttttcctgtcatactttgttaagaagggtgagaacagagtacctacattttgaatggaaggattggagctacgggggtgggggtggggtgggattagataaatgcctgctctttactgaaggctctttactattgctttatgataatgtttcatagttggatatcataatttaaacaagcaaaaccaaattaagggccagctcattcctcccactcatgatctatagatctatagatctctcgtgggatcattgtttttctcttgattcccactttgtggttctaagtactgtggtttccaaatgtgtcagtttcatagcctgaagaacgagatcagcagcctctgttccacatacacttcattctcagtattgttttgccaagttctaattccatcagaagctggtcgagatccggaacccttaatataacttcgtataatgtatgctatacgaagttattaggtccctcgaagaggttcactagtactggccattgcggccatcTTTAGATCCCGGGATGGCATTCCTGAGAAAGCAACCCGAACCAGCTGTCCTTCTGACAGCTCGGTGTTCAGCTTACAGAGACCACCCCTTTCCTCCACAGGGAGAGGCTCCTCCCTCTCCTGGGACAGCTCACAGGTCCTAGTGATTCTCTCAACCCTAACACCGTCTGGCACGATTGTGACCGCTGAAGTACACCACGAGCTCCAGGCTTCAGAAAGTGGTGCTGAGAACTTGCTCCAAGAAGAAGTCAAACCAAACTTGCAGTTGATTTGGGGTCATGTTTAGGTCAGAATCACCGTGCCCTTGAACAAGCAGGTAGGGGGGCTTGATAACTTAACTTTCCACGTGGACAGAATTTTTTTTTTTGTTTTGTTTTTGTTTTGCAGACACAGTCTAGCTCTGTCGCCCAGGCTGGAGTGCAGTGGCACGATCTCAGCTCACTGCAAGCTCTACCTCCCGGGTTCACGCCATTCTCCTGCCTCAGCCTCCCGAGTAGCTGGGACTACAGATGCCCACCACCAGGCCCGGCTAATTTTTTTTGTATTTTTAGTAGAGACGGGGTTTCACCGTGTTAGCCAGGATGGTCTCGATCTCCTGACCTCGTGATCCGCCCGCCTCGGCCTCCCAAAGTGCTAGGATTACAGGCGTGAGCCACCGCGCCCGGCCCTTTTTTTTTTTTTTTTTTTTAATTGAGACGGAGTCTCACTCTTTTGCTTAGGCTGGAGTGCAGTGGTGTGATCTCAGCTCACTGACTGCAACCTCCACCTCCCGGGTTGAAGCGTTGCTCCTGCCTCAATCTCC

**Key:**

5’ and 3’ CRX Homology Arms

eGFP

PGK Promoter

Puromycin
