# Supplementary material for: Using Zinc Finger Nuclease Technology to Generate CRX‐Reporter Human Embryonic Stem Cells as a Tool to Identify and Study the Emergence of Photoreceptors Precursors During Pluripotent Stem Cell Differentiation
Source: Stem Cells. 2015 Nov 26;34(2):311–21. doi: 10.1002/stem.2240 (PMC4832345; doi:10.1002/stem.2240)
Supplement: Supplementary file 5 — Supporting Information Table 1 [file STEM-34-311-s005.docx]

| **Supplementary Table 1: ZFN target sites and primers used in this study** | | |
| --- | --- | --- |
|  |  |  |
| ZFN Target Sites | Left | CTGAATCTGCTTCCCTGC |
|  | Right | AGATCCCGGGATGGC |
| Primers Flanking ZFN Target Site | Forward | GGCACCTGGAAATTCACCTA |
|  | Reverse | CCACTTTCTGAAGCCTGGAG |
| Primers Flanking Integration Site | Forward | CCCACAGCTGGATGCAAAGT |
|  | Reverse | AGATGGAACAGGCAAGGTGC |
| GFP Sequencing Primer |  | TTACGTCGCCGTCCAGCTC |
| Puromycin Sequencing Primer |  | GCATGGCCGAGTTGAGCGGT |
| Copy Number TaqMan Assays | EGFP | Mr00660654_cn |
|  | RNase P | 4403326 |
| Gene Expression TaqMan Assays | CRX | Hs00230899_m1 |
|  | EGFP | Mr04329676_mr |
|  | RHO | Hs00892431_m1 |
|  | NRL | Hs00172997_m1 |
|  | RCVRN | Hs00610056_m1 |
|  | ARR3 | Hs01020134_m1 |
|  | OPN1SW | Hs00181790_m1 |
|  | OPN1MW | Hs04194752_g1 |
|  | OPN1LW | Hs01912094_s1 |
|  | GAPDH | Hs99999905_m1 |
|  | MRPL19 | Hs00608519_m1 |
|  | RPLP0 | Hs99999902_m1 |
|  |  |  |
| **Collin et al. 2015** |  |  |
