# Supplementary material for: Using Zinc Finger Nuclease Technology to Generate CRX‐Reporter Human Embryonic Stem Cells as a Tool to Identify and Study the Emergence of Photoreceptors Precursors During Pluripotent Stem Cell Differentiation
Source: Stem Cells. 2015 Nov 26;34(2):311–21. doi: 10.1002/stem.2240 (PMC4832345; doi:10.1002/stem.2240)
Supplement: Supplementary file 7 — Supporting Information Table 3 [file STEM-34-311-s007.doc]

**Supplementary Table 3: Antibodies used for immunocytochemistry** in this study.

| **Antibody** | **Specificity** | **Host** | **Dilution** | **Supplier, Cat. No.** |
| --- | --- | --- | --- | --- |
| anti-OCT4 | pluripotent stem cells | rabbit | 1:100 | Life Technologies, A24867 |
| anti-SSEA4 | pluripotent stem cells | mouse | 1:100 | Life Technologies, A24866 |
| anti-TUJ1 | ectoderm (beta‑III tubulin) | rabbit | 1:500 | Life Technologies, A25532 |
| anti-AFP | endoderm (alpha-fetoprotein) | mouse | 1:500 | Life Technologies, A25530 |
| anti-SMA | mesoderm (smooth muscle actin) | mouse | 1:100 | Life Technologies, A25531 |
| anti-Calbindin D-28K (Calbindin 28) | horizontal cells, cone photoreceptors (excluding S cones), ON cone bipolar cells, wide field amacrine cells, large ganglion cells | rabbit | 1:500 | Chemicon, AB1778 |
| anti-Crx | postmitotic photoreceptors | mouse | 1:200 | Abnova, H00001406-M02 |
| anti GFP (biotin conjugate) | native GFP, GFP variants, and most GFP fusion proteins | chicken | 1:150 | Invitrogen, A10263 |
| anti-GFP (Alexa Fluor 488 conjugate) | native GFP, GFP variants, and most GFP fusion proteins | rabbit | 1:400 | Invitrogen, A21311 |
| anti-HuC/D | amacrine and ganglion cells | mouse | 1:200 | Molecular Probes, A21271 |
| anti-Ki67 | nuclear expression in proliferative cells during late G1, S, G2 and M phases of the cell cycle | rabbit | 1:200 | AbCam, Ab15580 |
| anti-Opsin blue | S cone photoreceptors | rabbit | 1:200 | Millipore, AB5407 |
| anti-Opsin red/green | L/M cone photoreceptors | rabbit | 1:200 | Millipore, AB5405 |
| anti-PAX6 | neural progenitors, retinal progenitors | rabbit | 1:300 | Covance, PRB-278P |
| anti-PKCα | rod ON bipolar cells | mouse | 1:300 | BD Transduction Laboratories, 610107 |
| anti-RAX | retina and anterior neural fold homeobox | rabbit | 1:200 | Aviva Systems Biology, ARP31926 |
| anti-Recoverin | photoreceptors and midget cone bipolar cells | rabbit | 1:500 | Chemicon, AB5585 |
