# Supplementary material for: Using Zinc Finger Nuclease Technology to Generate CRX‐Reporter Human Embryonic Stem Cells as a Tool to Identify and Study the Emergence of Photoreceptors Precursors During Pluripotent Stem Cell Differentiation
Source: Stem Cells. 2015 Nov 26;34(2):311–21. doi: 10.1002/stem.2240 (PMC4832345; doi:10.1002/stem.2240)
Supplement: Supplementary file 9 — Supporting Information [file STEM-34-311-s009.doc]

**Supplemental Figure Legends and Tables Information – Lako et al.**

**Suppl. Figure 1: Surveyor mutation detection assay performed in K562 cells and hESCs to indicate ZFN cutting efficiency**. As shown, a higher ZFN cutting efficiency is observed in K562 cells than hESCs (which may be related to lower non homologous end joining repair pathway efficiency observed in hESCs). Upon cleavage of the target site by the ZFN pair inaccurate repair by the non-homologous end joining pathway can occur resulting in insertion or deletion of nucleotides. Following PCR of the target region and cooling of the PCR products duplexes form. In the presence of NHEJ-repaired and wild-type heteroduplexes and the SURVEYOR/CEL-I enzyme cleavage products are produced. The 221 and 150 bp bands represent the cleavage of PCR products generated across the *CRX* 3’ UTR target cut site in the presence of an insertion or deletion, in the absence of an insertion or deletion a wild-type PCR product of 371 bp is observed. (**A**) K562 cells; lane 1, PCR of wild-type genomic DNA (No), lanes 2 & 3, PCR of genomic DNA following ZFN pair transfection (ZFN); (**B**) hESC H9 cells; lane 1, PCR of wild-type genomic DNA (No), lane 2, PCR of genomic DNA following a mock nucleofection without ZFNs (Mock) & lane 3, PCR of genomic DNA following ZFN pair nucleofection (ZFN).

**Suppl. Figure 2: The sequence of CRX-GFP targeting construct.** The sequences of *CRX* 5’ and 3’ homology arms, eGFP reporter and puromycin resistance cassette are shown as highlighted in the key.

**Suppl. Figure 3: CRX-GFP targeted hESC clones maintain a normal karyotype and exhibit pluripotency.** (**A**) Normal karyotype (46 XX) observed in CRX-GFP hESC clone 1; (**B**) Pluripotent marker staining (OCT4 and SSEA4) in CRX-GFP hESC clone 1, scale bars = 200 µm; (**C**) Three germ layer differentiation capacity assessed by the presence of endodermal cells (marked by AFP staining), ectodermal cells (marked by TUJ1 staining) and mesodermal cells (marked by SMA) in embryoid bodies derived from differentiation of CRX-GFP hESC clone 1, scale bars = 100 µm; (**D**) Histological analysis of xenograft tumours formed from engrafted CRX-GFP hESC clone 3. The teratomae produced contained tissues representative of endoderm (***a***), mesoderm (***b***) and ectoderm (***c***). Example tissues included: (***a***) structure of primitive intestine showing epithelium (ep), villous (vi), submucosa (sb), and smooth muscle (sm); (***b***) cartilaginous masses (ct); (***c***) neuro-epithelium (ne). Counterstain: Haematoxylin and Eosin. Scale bars (a-c) = 150 µm.

**Suppl. Figure 4: CRX is not expressed in early stages of eye cup formation**. Immunocytochemistry with antibodies raised against GFP, RAX (**A**) and PAX6 (**B**) (both markers of eyefield development) at day 60 of differentiation. Scale bars = 20 µm.

**Suppl. Figure 5: CRX and RECOVERIN expression during early human fetal development at 16 (A) and 18 weeks of gestation (B-D)**. Double positive CRX and RECOVERIN expressing cells are located in the developing outer nuclear layer. In addition CRX+RECOVERIN- cells are found in the neuroblastic layer. Scale bars = 100 µm (A) and 50 µm (B-D).

**Suppl. Table 1: ZFN pair target sites, PCR primers, sequencing primers.**

**Suppl. Table 2: ZFN pair off-target analysis. Off-target site sequences, primer details and sequencing.**

**Suppl. Table 3: Antibodies used during this study.**
